# Stress, anxiety, and depression in infertile couples are not associated with a first IVF or ICSI treatment outcome

**DOI:** 10.1186/s12884-021-04202-9

**Published:** 2021-10-27

**Authors:** Meijuan Peng, Mingyang Wen, Tao Jiang, Yangqian Jiang, Hong Lv, Ting Chen, Xiufeng Ling, Hong Li, Qingxia Meng, Boxian Huang, Shiyao Tao, Lei Huang, Cong Liu, Xin Xu, Qun Lu, Xiaoyu Liu, Bo Xu, Xiumei Han, Kun Zhou, Jiaping Chen, Yuan Lin, Hongxia Ma, Yankai Xia, Hongbing Shen, Zhibin Hu, Feng Chen, Jiangbo Du, Guangfu Jin

**Affiliations:** 1 Department of Epidemiology, Center for Global Health, School of Public Health, Nanjing Medical University, Nanjing, 211166 Jiangsu China; 2 State Key Laboratory of Reproductive Medicine, Nanjing Medical University, Nanjing, 211166 Jiangsu China; 3 Department of Biostatistics, School of Public Health, Nanjing Medical University, Nanjing, 211166 Jiangsu China; 4 Department of Science and Technology, Women’s Hospital of Nanjing Medical University, Nanjing Maternity and Child Health Care Hospital, Nanjing, 210004 Jiangsu China; 5 Department of Reproduction, Women’s Hospital of Nanjing Medical University, Nanjing Maternity and Child Health Care Hospital, Nanjing, 210004 Jiangsu China; 6 Reproductive Genetic Center, The Affiliated Suzhou Hospital of Nanjing Medical University, Suzhou Municipal Hospital, Gusu School, Nanjing Medical University, Suzhou, 215002 Jiangsu China; 7 Department of Maternal, Child and Adolescent Health, School of Public Health, Nanjing Medical University, Nanjing, 211166 Jiangsu China; 8 Key Laboratory of Modern Toxicology of Ministry of Education, School of Public Health, Nanjing Medical University, Nanjing, 211166 Jiangsu China

**Keywords:** Intracytoplasmic sperm injection (ICSI), In vitro fertilization (IVF), Psychological distress, Clinical pregnancy

## Abstract

**Background:**

Psychological distress may exert a negative influence on reproductive function of couples at reproductive age. Couples seeking assisted reproductive technology (ART) treatment may have a higher prevalence of psychological distress than fertile couples. However, whether psychological distress is associated with the outcome of ART treatment remains unknown. We aimed to investigate the association of pre-treatment psychological distress and clinical pregnancy rate among infertility couples undergoing in vitro fertilization (IVF) or intracytoplasmic sperm injection (ICSI) treatment.

**Methods:**

This nested case-control study was conducted based on women who underwent their first fresh IVF or ICSI cycle in the Jiangsu Birth Cohort Study (JBC) between November 2015 and January 2019. A total of 150 women who did not obtain clinical pregnancy after first IVF or ICSI fresh embryo transfer were identified as cases, and a total of 300 age matched women who obtained clinical pregnancy were identified as controls. Conditional logistic regression analyses were used to investigate the association between psychological distress and the outcome of first IVF or ICSI treatment, adjusting for multiple potential confounders.

**Results:**

No statistically significant association was observed between score of maternal symptoms of psychological distress and clinical pregnancy. Adjusted ORs of logistic regression were 1.00 (95% CI 0.97-1.03) for anxiety, 0.98 (95% CI 0.95-1.02) for depression, and 0.98 (95% CI 0.95-1.01) for perceived stress, respectively. When treat depression and anxiety as categorical variables, 62 (13.8%) were classified as clinical depression, 11 (2.4%) were classified as clinical anxiety, among 450 women in the present study. Psychological distress symptoms were also not associated with clinical pregnancy rate. Adjusted ORs of logistic regression were 0.27 (95% CI 0.03-2.33) for anxiety, 0.88 (95% CI 0.46-1.68) for depression, respectively.

**Conclusions:**

Our findings firstly indicated that psychological distress experienced prior to IVF/ICSI treatment was not associated with clinical pregnancy.

**Supplementary Information:**

The online version contains supplementary material available at 10.1186/s12884-021-04202-9.

## Background

During the past few decades, assisted reproductive technology (ART) is widely practiced throughout the world. However, the rate of clinical pregnancy is still low [1]. Inadequate ovarian reserve [2], the presence of hydrosalpinx [3], uterine myoma [4], and endometriosis [5, 6] have been established as the main pathological factors, but the determinants of clinical pregnancy is still not fully revealed. Recently, many have shown that psychological distress may aggravate poor fertility [7, 8]. Pathways of the hypothalamic-pituitary adrenal axis or the hypothalamic-pituitary gonadal axis may play a key role in this regulation process [9–11]. Couples seeking ART treatment may have a higher prevalence of psychological distress than fertile couples [12], because of the permanency of infertility, loss of hope, the treatment itself, and several previous ART attempts [13–15]. Therefore, whether psychological distress is associated with the outcome of ART treatment has aroused widely concern.

Relationship between psychological distress and the outcome of ART treatment remains inconclusive [16–20]. Three studies have reported that psychological distress was associated with decreased pregnancy rates in in vitro fertilization (IVF) patients [16, 18, 21]. In contrast, a recent meta-analysis found that baseline (before ART treatment has started) psychological distress was not associated with ART outcome [22]. In addition, psychological distress covers a variety of symptoms [23, 24], but most previous studies only focused on single psychological distress [21, 25–28]. Furthermore, most of these studies only included IVF cycle data, and could not extend their findings to patients with intracytoplasmic sperm injection (ICSI) treatment [14, 16, 18, 21, 25, 29, 30]. Therefore, we conducted this nested case-control study based on a large prospective multicenter cohort in Chinese population, aimed to investigate the association of pre-treatment psychological distress and clinical pregnancy rate among infertility couples undergoing IVF or ICSI treatment.

## Methods

### Study population

We conducted this nested case-control study within the Jiangsu Birth Cohort Study (JBC), a prospective and longitudinal birth cohort study. The recruitment and assignment of JBC has been described in previous study [31]. Briefly, JBC recruited couples who were about to receive assisted reproduction at the Women’s Hospital of Nanjing Medical University or Suzhou Affiliated Hospital of Nanjing Medical University. They completed standardized and structured questionnaires by face-to-face interview to collect their demographic information. The JBC followed up both of assisted reproductive outcomes and obstetrics outcome using data from medical records and questionnaires. In this study, we identified women who underwent their first fresh IVF or ICSI cycle between November 2015 and January 2019. Women who have a history of three or more pregnancy losses were excluded from this study. A total of 150 women who did not obtain clinical pregnancy after first IVF or ICSI fresh embryo transfer were identified as cases, and a total of 300 age matched women who obtained clinical pregnancy were identified as controls. Clinical pregnancy was defined as the presence of one or more intrauterine gestational sacs with normal cardiac activity.

Cleavage-stage embryos were graded in three categories (Good, Fair and Poor) according to “ASEBIR embryo assessment criteria” [32], taking into account seven parameters (Day, Cell number, Fragmentation, Symmetry, Multi-nucleation, Vacuoles, Zona pellucida). Blastocysts were divided into three groups based on “consensus scoring system for blastocysts” [32], according to the stage of development, the morphologic grade of the inner cell mass and the trophectoderm: Good (1-6AA, 3-6AB, 3-6BA), Fair (3-6BB, 3-6 AC, 3-6CA, 1-2AB, 1-2BA), and Poor (1-6 BC, 1-6CB, 1-6CC, 1-2BB). Each grade is given a score (3 = good, 2 = fair, and 1 = poor) as the embryo score. The embryo scores of all transferred embryos are added together to obtain the embryo sum score.

The controlled ovarian hyperstimulation (COH) protocol was divided into three groups, including agonists protocol, antagonist protocol and other protocols, based on the usage of a gonadotropin-releasing hormone agonist (GnRH-a) versus antagonist analog. In the GnRH-a protocol, GnRH-a was used in the mid-luteal phase of the first menstrual cycle. Fourteen days later, exogenous gonadotropin (Gn), including FSH, luteinizing hormone (LH) and human menopausal gonadotropin (hMG) was used to promote ovulation when the pituitary reached the regulation standard. Human chorionic gonadotropin (HCG) was injected in the case of three or more follicles with a diameter of 16 ~ 18 mm and the oocytes were taken after 36 h. In the GnRH antagonist protocols, Gn was used on 2 ~ 3 days of the menstrual cycle. When the dominant follicle reached 12 ~ 14 mm or LH > 10 U/L, antagonists were used. HCG was injected when there were three or more follicles with diameters of 16 ~ 18 mm, and oocytes were taken after 36 h.

All methods and protocols for information collection were approved by the institutional review board of Nanjing Medical University, China NJMUIRB (2017) 002. The recruitment performed in accordance to the Helsinki declaration. Informed, written consent was obtained from all participants.

### Psychological assessment

Psychological distress of couples including anxiety, depression and perceived stress were assessed before the assisted reproductive treatment. Anxiety was measured with the Self-Rating Anxiety Scale (SAS) [33]. The scale comprises 20 items covering autonomic, cognitive, motor, and central nervous systems symptoms. Each item is scored on a Likert scale ranging from 1 to 4 (1 = none or a little of the time, 2 = some of the time, 3 = good part of the time, 4 = most or all of the time). Participants with SAS standard scores ≥50 were considered at risk for clinical anxiety [34]. Depression was assessed using the Center for Epidemiologic Study of Depression Scale (CESD). The CESD consists of 20 items which are rated using a 4-point ordered response set to indicate how frequently symptoms were experienced during the previous week (0 = rarely or none of the time, 1 = some or a little of the time, 2 = occasionally or a moderate amount of the time, 3 = most or all of the time). Total score of CESD was generated by summing their item responses and ranging from 0 to 60 (higher scores indicating more depressive symptoms). Participants with CESD scores ≥16 were considered at risk for clinical depression [35]. Perceived stress was assessed with the Perceived Stress Scale (PSS-10), which consists of 10 items purported to measure the degree of nonspecific appraised stress over the past month [36]. Each item was rated using a 5-point ordered to indicate the frequently symptoms (0 = never, 1 = almost never, 2 = sometimes, 3 = fairly often, 4 = very often). The total PSS-10 score was ranging from 0 to 40, higher score represents greater stress [37].

### Covariate information

We selected several potential confounders as covariates by reviewing the literatures [38–43]. Information on female body mass index (BMI), female educational attainment (< 12 years, ≥12 years), female occupation (mental worker, physical worker or none), household income (< 50,000 CNY, 50000 ~ 100,000 CNY, 100000 ~ 200,000 CNY, > 200,000 CNY), female and male smoking (none versus any), alcohol use (rarely: < 1 time/month; regular: ≥1 time/month), sleep quality (good versus poor), and exercise (rarely: < 3 times/week; regular: ≥3 times/week) before the start of treatment were retrieved from the questionnaire data. Infertility factor (female factor, male factor, couple’s factor, and unexplained factor), duration of infertility, and prior history of pregnancy loss (nulliparous, gravid with no prior history of loss, gravid with prior history of loss) were retrieved from medical records. Sleep quality was assessed by the Pittsburgh Sleep Quality Index (PSQI) developed by Buysse et al. [44]. It is a self-rated questionnaire and disturbances over an l-month time interval while higher scores represent worse sleep quality [44]. We used the established cutoff > 5 to depict poor sleep quality [44, 45].

### Statistical analysis

Non-normally distributed variables were reported as the median (25th-75th range) and were compared using the Mann-Whitney U test among groups. Nominal variables were tested either with the Chi-square test or Fisher’s exact test. Conditional logistic regression was used to estimate ORs with 95% CIs to assess the association between pre-treatment psychological distress and clinical pregnancy. All statistical analyses were performed using the R software version 4.0.2 (http://www.R-project.org/). *P* < 0.05 was considered statistically significant.

## Results

As shown in Table 1, a total of 150 cases of women that failed to obtained clinical pregnancy and 300 controls of women that obtained clinical pregnancy were included in this study. The age was adequately matched between cases and controls (*P* > 0.05). Similar distributions of other baseline characteristics were also observed between cases and controls. Infertility factors were not associated to the clinical pregnancy (Supplementary Table 1).

**Table 1:** Characteristics of the study population (*N* = 450 women) undergoing their first recorded fresh IVF or ICSI cycle

Covariate	Pregnancy ***N*** = 300	Non-pregnancy ***N*** = 150	***P***
**Age at first cycle**^**a**^	31.0 (29.0, 33.0)	31.0 (29.0, 34.0)	0.123^c^
**BMI before transfer, kg/m**^**2**^**, No. (%)**			
< 18.5	19 (6.3)	9 (6.0)	0.954^e^
18.5 ≤ BMI < 24	210 (70.0)	102 (68.0)	
24 ≤ BMI < 28	58 (19.3)	32 (21.3)	
BMI ≥ 28	13 (4.3)	7 (4.7)	
**Annual Household Income, 10,000 CNY, No. (%)**			
< 5	12 (4.0)	7 (4.7)	0.423^e^
5 ~ 10	77 (25.7)	48 (32.0)	
10 ~ 20	118 (39.3)	49 (32.7)	
> 20	93 (31.0)	46 (30.7)	
**Occupation (%)**			
Mental worker	150 (50.0)	73 (48.7)	0.831^d^
Physical worker	135 (45.0)	71 (47.3)	
No	15 (5.0)	6 (4.0)	
**Education, year, No. (%)**			
≥ 12	218 (72.7)	103 (68.7)	0.439^d^
< 12	82 (27.3)	47 (31.3)	
**Prior history of pregnancy loss (%)**			
Gravid, with no prior history of loss	12 (4.0)	8 (5.3)	0.508^e^
Gravid, with prior history of loss	14 (4.7)	10 (6.7)	
Nulliparous	274 (91.3)	132 (88.0)	
**Sleep quality (%)**^**b**^			
Good	228 (77.6)	124 (85.5)	0.065^d^
Poor	66 (22.4)	21 (14.5)	
**Infertility factor (%)**			
Couple’s factor	181 (60.3)	99 (66.0)	0.399^e^
Female factor	98 (32.7)	46 (30.7)	
Male factor	19 (6.3)	5 (3.3)	
Unexplained factor	2 (0.7)	0 (0.0)	
**Duration of infertility (m)**	36.0 (24.0, 53.0)	36.0 (24.0, 60.0)	0.683^c^
**Smoking before transfer, No. (%)**			
Non-smoker	297 (99.0)	147 (98.0)	0.405^e^
Smoker	3 (1.0)	3 (2.0)	
**Alcohol use before transfer, No. (%)**			
Rarely	292 (97.3)	148 (98.7)	0.507^e^
Regular	8 (2.7)	2 (1.3)	
**Exercise, No. (%)**			
Rarely	249 (83.0)	124 (82.7)	1.000^d^
Regular	51 (17.0)	26 (17.3)	

Values are number of women (%) for categorical variables, median and range for continuous variables unless indicated otherwise

^a^Cases and controls were matched on female age

^b^Variable contains missing data

^c^*P* values were derived with Mann–Whitney U test for nonnormally distributed continuous variables

^d^*P* values were derived with Pearson chi-square test for categorical variables

^e^*P* values were derived with Fisher exact test for categorical variables with less than 10 observations per category

Clinical pregnant women shown similar median score compared with non-pregnant women (SAS score of women: 31.0 versus 32.0 points, *P* = 0.761; CESD score of women: 6.0 versus 5.5 points, *P* = 0.387; PSS score of women: 11.0 versus 10.0 points, *P* = 0.208). Among their partners, there were no differences in the median anxiety, depression and perceived stress levels between the two groups. Partners of pregnant women also have a similar median score compared with those of non-pregnant women (SAS score of partners: 28.0 versus 28.0 points, *P* = 0.859; CESD score of partners: 4.0 versus 4.0 points, *P* = 0.674; PSS score of partners: 10.0 versus 9.0 points, *P* = 0.463). Pregnant couples did have a similar median score compared with non-pregnant couples (SAS score of couples: 61.0 versus 61.0 points, *P* = 0.91; CESD score of couples: 12.0 versus 11.0 points, *P* = 0.685; PSS score of couples: 20.5 versus 20.0 points, *P* = 0.165). Logistic regression analyses showed that continuous pre-treatment psychological distress score were not associated with clinical pregnancy outcome of the first fresh cycle (Table 2). Adjusted ORs of were 1.00 (95% CI 0.97-1.03) for anxiety, 0.98 (95% CI 0.95-1.02) for depression, and 0.98 (95% CI 0.95-1.01) for perceived stress, respectively. Similar associations were observed in their partners and in couples (Table 2).

**Table 2:** Conditional logistic Regression Analysis of psychological distress level on pregnancy rate of first IVF or ICSI cycle among 450 couples

Psychological distress	Pregnancy	Non-pregnancy	***P***^***a***^	Crude OR^**b**^ (95% CI)	Adjusted OR^**c**^ (95% CI)	Adjusted OR^**d**^ (95%CI)
Woman						
Anxiety	31.0 (26.0, 36.3)	32.0 (26.0, 37.0)	0.761	1.00 (0.97-1.03)	1.00 (0.97-1.03)	1.00 (0.97-1.03)
Depression	6.0 (1.8, 12.0)	5.5 (1.0, 11.0)	0.387	0.98 (0.95-1.01)	0.98 (0.95-1.01)	0.98 (0.95-1.02)
Perceived stress	11.0 (4.0, 15.0)	10.0 (2.0, 14.0)	0.208	0.98 (0.95-1.01)	0.98 (0.95-1.01)	0.98 (0.95-1.01)
Partner ^e^						
Anxiety	28.0 (25.0, 33.0)	28.0 (25.0, 33.0)	0.859	0.99 (0.95-1.02)	0.98 (0.95-1.01)	0.98 (0.95-1.02)
Depression	4.0 (0.0, 9.0)	4.0 (0.0, 10.0)	0.674	1.00 (0.97-1.02)	0.99 (0.96-1.02)	0.99 (0.96-1.03)
Perceived stress	10.0 (3.0, 14.0)	9.0 (1.5, 15.0)	0.463	0.99 (0.96-1.02)	0.98 (0.95-1.01)	0.99 (0.96-1.02)
Couple ^e^						
Anxiety	61.0 (53.0, 68.0)	61.0 (53.0, 68.0)	0.910	0.99 (0.97-1.01)	0.99 (0.97-1.01)	0.99 (0.97-1.01)
Depression	12.0 (3.0, 20.0)	11.0 (3.0, 20.8)	0.685	0.99 (0.97-1.01)	0.99 (0.97-1.00)	0.99 (0.97-1.01)
Perceived stress	20.5 (8.0, 29.0)	20.0 (6.0, 27.0)	0.165	0.99 (0.97-1.00)	0.98 (0.97-1.00)	0.99 (0.97-1.01)

Values are median and range for continuous variables unless indicated otherwise

^a^*P* values were derived with Mann–Whitney U test for nonnormally distributed continuous variables

^b^Univariable conditional logistic regression analyses of psychological distress level on pregnancy rate of first IVF or ICSI cycle

^c^Model 1: Multivariable conditional logistic regression analyses were adjusted for female pre-treatment BMI, educational attainment, occupation, household income, infertility factor, duration of infertility

^d^Model 2: Multivariable conditional logistic regression analyses were adjusted for female pre-treatment BMI, educational attainment, occupation, household income, infertility factor, duration of infertility, prior history of pregnancy loss, alcohol use, sleep quality, exercise, female and male smoking before the start of treatment

^e^Variable contains missing data

When treat depression and anxiety as categorical variables, 62 (13.8%) were classified as clinical depression, 11 (2.4%) were classified as clinical anxiety, among 450 women in the present study. Psychological distress symptoms were also not associated with clinical pregnancy rate. Adjusted ORs of logistic regression were 0.27 (95% CI 0.03-2.33) for anxiety, 0.88 (95% CI 0.46-1.68) for depression, respectively. Furthermore, using women without any psychological symptom (neither depression nor anxiety) as a reference, the ORs for were 0.95 (95% CI 0.49-1.84) for those who exposed to one symptom (anxiety or depression), and 0.29 (95% CI 0.03-2.63) for two symptoms (anxiety and depression), respectively (Table 3).

**Table 3:** Conditional logistic Regression Analysis of psychological distress symptoms on pregnancy rate of first IVF or ICSI cycle among 450 women

Psychological distress	N	No. of non-pregnancy (%)	Crude OR^**a**^ (95%CI)	Adjusted OR^**b**^ (95%CI)	Adjusted OR^**c**^ (95%CI)
**Anxiety**					
No	439	148 (33.7)	1.00	1.00	1.00
Yes	11	2 (18.2)	0.44 (0.10-2.06)	0.46 (0.10-2.20)	0.27 (0.03-2.33)
**Depression**					
No	388	129 (33.2)	1.00	1.00	1.00
Yes	62	21 (33.9)	1.03 (0.57-1.85)	0.93 (0.51-1.70)	0.88 (0.46-1.68)
**Psychological distress**^d^					
Norm	387	129 (33.3)	1.00	1.00	1.00
One symptom (Depression/Anxiety)	53	19 (35.8)	1.11 (0.60-2.06)	0.99 (0.53-1.85)	0.95 (0.49-1.84)
Two symptoms (Depression and Anxiety)	10	2 (20.0)	0.51 (0.11-2.42)	0.52 (0.11-2.54)	0.29 (0.03-2.63)

^a^Univariable conditional logistic regression analyses of psychological distress level on pregnancy rate of first IVF or ICSI cycle

^b^Model 1: Multivariable conditional logistic regression analyses were adjusted for female pre-treatment BMI, educational attainment, occupation, household income, infertility factor, duration of infertility

^c^Model 2: Multivariable conditional logistic regression analyses were adjusted for female pre-treatment BMI, educational attainment, occupation, household income, infertility factor, duration of infertility, prior history of pregnancy loss, alcohol use, sleep quality, exercise, female and male smoking before the start of treatment

^d^Norm was defined as women without any symptom including depression, anxiety. One symptom was defined as women with any symptom including depression or anxiety. Two symptoms were defined as women with depression and anxiety

## Discussion

In this nested case-control study, we found that psychological distress before IVF or ICSI treatment, in general or as specific types, were not associated with clinical pregnancy in infertile couples during the first fresh cycle. This study is the first one to evaluate the effect of pre-pregnancy depression, anxiety, or stress individually and comprehensively on the clinical pregnancy probability among IVF/ICSI treated women of infertility in the Chinese population.

Our finding was supported by several recent studies [14, 26–29, 46–48]. Three prospective studies of the literature on stress and IVF outcome had concluded that stress in women, before or during treatment, was not correlated with pregnancy outcome [26–28]. Concerning to depression and anxiety, four prospective studies had concluded that no association between pre-treatment depression/anxiety in women and pregnancy outcome [14, 29, 46, 48]. In addition, only one study in Chinese population (264 IVF or ICSI women) had explored depression, anxiety and stress simultaneously, which reported that women’s stress, anxiety, and depression were unlikely have correlation with clinical pregnancy [29]. Given most of previous studies only focused on single aspect of psychological distress and lack of control for potential confounders, our study provided more reliable evidence for the association.

Some studies showed that psychological distress predicted a higher rate of poor outcomes [25, 49–51]. However, there were several limitations should be concerned. First, most of the former studies did not included women with only first-time fresh IVF or ICSI. Because, women’s emotional experiences might be affected by previous experience of ART treatment [13, 14, 52, 53]. Second, few studies used multidimensional evaluation of psychological distress of in infertile couples. Third, a majority of studies couldn’t were failed to fully control for potential confounding factors for the association, such as lifestyle factors, duration and cause of infertility, pregnancy history [38–40, 42, 54, 55]. Furthermore, sleep quality was not taken into consideration in previous studies [56–58].

The main strengths of our study include the prospective cohort based nested case-control design and standard assessments conducted separately in two Chinese ART clinics. In addition, our data are available to adjust for potential confounders such as causes of infertility and important lifestyle factors. Some limitations should be also noted. First, although our perceived psychological scales were simultaneously administered before treatment, but single time point limited us to comprehensively evaluate the psychological influence on outcome of ART treatment. Second, the data of psychotherapy or psychopharmacological treatments was not available, thus we could not control the potential confounding on our findings. Third, our sample size is slightly larger than two existing literature in China [27, 29], but still might be inadequate to detect relatively small effects. In addition, we included unequal groups (150 cases versus 300 controls) in our study, which may lead to additional bias [59]. Therefore, validation study with larger sample size is warranted in the future.

## Conclusion

In summary, our study on psychological distress and IVF or ICSI outcome did not observed significant influence of pre-treatment psychological distress (e.g., anxiety or depression) on the rate of clinical pregnancy. Further, women are still encouraged to express psychological distress before treatment and the development of intervention strategies to improve coping are helpful, not only toward reducing emotional suffering, but also to avoid discontinuing treatment before reaching goal of live birth.

## Supplementary Information

**Additional file 1: Supplementary Table 1.** Characteristics of the ART procedures among 450 couples undergoing their first recorded fresh IVF or ICSI cycle. **Supplementary Table 2.** Conditional logistic Regression Analysis of psychological distress level on pregnancy rate of first IVF or ICSI cycle among couples without endometriosis, or chronic endometritis or autoimmune disorders. **Supplementary Table 3.** Conditional logistic Regression Analysis of psychological distress symptoms on pregnancy rate of first IVF or ICSI cycle among women without endometriosis, or chronic endometritis or autoimmune disorders. **Supplementary Figure 1.** Flowchart for inclusion and exclusion of the study population.

## Data Availability

The datasets during and/or analyzed during the current study available from the corresponding author on reasonable request.

## References

[1] Adamson GD, de Mouzon J, Chambers GM, Zegers-Hochschild F, Mansour R, Ishihara O, Banker M, Dyer S (2018). International Committee for Monitoring Assisted Reproductive Technology: world report on assisted reproductive technology, 2011. Fertil Steril.

[2] Gleicher N, Kushnir VA, Sen A, Darmon SK, Weghofer A, Wu YG, Wang Q, Zhang L, Albertini DF, Barad DH (2016). Definition by FSH, AMH and embryo numbers of good-, intermediate- and poor-prognosis patients suggests previously unknown IVF outcome-determining factor associated with AMH. J Transl Med.

[3] Schlaff WD (2019). A reconsideration of salpingectomy for hydrosalpinx before in vitro fertilization: why bother?. Fertil Steril.

[4] Cook H, Ezzati M, Segars JH, McCarthy K (2010). The impact of uterine leiomyomas on reproductive outcomes. Minerva Ginecol.

[5] Vercellini P, Consonni D, Dridi D, Bracco B, Frattaruolo MP, Somigliana E (2014). Uterine adenomyosis and in vitro fertilization outcome: a systematic review and meta-analysis. Hum Reprod.

[6] Salim R, Riris S, Saab W, Abramov B, Khadum I, Serhal P (2012). Adenomyosis reduces pregnancy rates in infertile women undergoing IVF. Reprod BioMed Online.

[7] Sominsky L, Hodgson DM, McLaughlin EA, Smith R, Wall HM, Spencer SJ (2017). Linking stress and infertility: a novel role for ghrelin. Endocr Rev.

[8] Lynch CD, Sundaram R, Maisog JM, Sweeney AM, Buck Louis GM (2014). Preconception stress increases the risk of infertility: results from a couple-based prospective cohort study--the LIFE study. Hum Reprod.

[9] Toufexis D, Rivarola MA, Lara H, Viau V (2014). Stress and the reproductive axis. J Neuroendocrinol.

[10] Massey AJ, Campbell BK, Raine-Fenning N, Pincott-Allen C, Perry J, Vedhara K (2016). Relationship between hair and salivary cortisol and pregnancy in women undergoing IVF. Psychoneuroendocrinology.

[11] Joseph DN, Whirledge S (2017). Stress and the HPA Axis: Balancing Homeostasis and Fertility. Int J Mol Sci.

[12] Verhaak CM, Smeenk JM, Evers AW, Kremer JA, Kraaimaat FW, Braat DD (2007). Women's emotional adjustment to IVF: a systematic review of 25 years of research. Hum Reprod Update.

[13] Verhaak CM, Smeenk JM, van Minnen A, Kremer JA, Kraaimaat FW (2005). A longitudinal, prospective study on emotional adjustment before, during and after consecutive fertility treatment cycles. Hum Reprod.

[14] Pasch LA, Gregorich SE, Katz PK, Millstein SG, Nachtigall RD, Bleil ME, Adler NE (2012). Psychological distress and in vitro fertilization outcome. Fertil Steril.

[15] Nicoloro-SantaBarbara JM, Lobel M, Bocca S, Stelling JR, Pastore LM (2017). Psychological and emotional concomitants of infertility diagnosis in women with diminished ovarian reserve or anatomical cause of infertility. Fertil Steril.

[16] Cesta CE, Viktorin A, Olsson H, Johansson V, Sjölander A, Bergh C, Skalkidou A, Nygren KG, Cnattingius S, Iliadou AN (2016). Depression, anxiety, and antidepressant treatment in women: association with in vitro fertilization outcome. Fertil Steril.

[17] Lynch CD, Sundaram R, Buck Louis GM (2018). Biomarkers of preconception stress and the incidence of pregnancy loss. Hum Reprod.

[18] Domar AD, Gross J, Rooney K, Boivin J (2015). Exploratory randomized trial on the effect of a brief psychological intervention on emotions, quality of life, discontinuation, and pregnancy rates in in vitro fertilization patients. Fertil Steril.

[19] Bapayeva G, Aimagambetova G, Issanov A, Terzic S, Ukybassova T, Aldiyarova A, Utepova G, Daribay Z, Bekbossinova G, Balykov A (2021). The Effect of Stress, Anxiety and Depression on In Vitro Fertilization Outcome in Kazakhstani Public Clinical Setting: A Cross-Sectional Study. J Clin Med.

[20] Maroufizadeh S, Navid B, Omani-Samani R, Amini P (2019). The effects of depression, anxiety and stress symptoms on the clinical pregnancy rate in women undergoing IVF treatment. BMC Res Notes.

[21] Louis GM, Lum KJ, Sundaram R, Chen Z, Kim S, Lynch CD, Schisterman EF, Pyper C (2011). Stress reduces conception probabilities across the fertile window: evidence in support of relaxation. Fertil Steril.

[22] Purewal S, Chapman SCE, van den Akker OBA (2018). Depression and state anxiety scores during assisted reproductive treatment are associated with outcome: a meta-analysis. Reprod BioMed Online.

[23] Ridner SH (2004). Psychological distress: concept analysis. J Adv Nurs.

[24] Masse R (2000). Qualitative and quantitative analyses of psychological distress: methodological complementarity and ontological incommensurability. Qual Health Res.

[25] Zhou FJ, Cai YN, Dong YZ (2019). Stress increases the risk of pregnancy failure in couples undergoing IVF. Stress.

[26] Miller N, Herzberger EH, Pasternak Y, Klement AH, Shavit T, Yaniv RT, Ghetler Y, Neumark E, Eisenberg MM, Berkovitz A (2019). Does stress affect IVF outcomes? A prospective study of physiological and psychological stress in women undergoing IVF. Reprod BioMed Online.

[27] Cheung C, Saravelos SH, Chan T, Sahota DS, Wang CC, Chung PW, Li TC (2019). A prospective observational study on the stress levels at the time of embryo transfer and pregnancy testing following in vitro fertilisation treatment: a comparison between women with different treatment outcomes. BJOG.

[28] Anderheim L, Holter H, Bergh C, Moller A (2005). Does psychological stress affect the outcome of in vitro fertilization?. Hum Reprod.

[29] An Y, Wang Z, Ji H, Zhang Y, Wu K (2011). Pituitary-adrenal and sympathetic nervous system responses to psychiatric disorders in women undergoing in vitro fertilization treatment. Fertil Steril.

[30] Rooney KL, Domar AD (2018). The relationship between stress and infertility. Dialogues Clin Neurosci.

[31] Lv H, Diao F, Du J, Chen T, Meng Q, Ling X, et al. Assisted reproductive technology and birth defects in a Chinese birth cohort study. Lancet Reg Health West Pac. 2021;7. https://www.thelancet.com/journals/lanwpc/article/PIIS2666-6065(20)30090-0/fulltext. 10.1016/j.lanwpc.2020.100090 PMC8315325 34327418

[32] Alpha Scientists in Reproductive Medicine and ESHRE Special Interest Group of Embryology. The Istanbul consensus workshop on embryo assessment: proceedings of an expert meeting. Hum Reprod. 2011;26(6):1270–83. 10.1093/humrep/der037 21502182

[33] Zung WW (1971). A rating instrument for anxiety disorders. Psychosomatics.

[34] Zung WW, Magruder-Habib K, Velez R, Alling W (1990). The comorbidity of anxiety and depression in general medical patients: a longitudinal study. J Clin Psychiatry.

[35] Roberts RE, Vernon SW (1983). The Center for Epidemiologic Studies Depression Scale: its use in a community sample. Am J Psychiatry.

[36] Cohen S, Williamson G. Perceived stress in a probability sample of the United States. In: Spacapan S, Oskamp S, editors. The Social Psychology of Health. Thousand Oaks: Sage Publications, Inc.; 1988. pp. 31–67.

[37] Cohen S, Kamarck T, Mermelstein R (1983). A global measure of perceived stress. J Health Soc Behav.

[38] Klonoff-Cohen H (2005). Female and male lifestyle habits and IVF: what is known and unknown. Hum Reprod Update.

[39] Sharpe RM, Franks S (2002). Environment, lifestyle and infertility--an inter-generational issue. Nat Cell Biol.

[40] Balchin R, Linde J, Blackhurst D, Rauch HL, Schonbachler G (2016). Sweating away depression? The impact of intensive exercise on depression. J Affect Disord.

[41] van der Steeg JW, Steures P, Eijkemans MJ, Habbema JD, Hompes PG, Michgelsen HW, van der Heijden PF, Bossuyt PM, van der Veen F, Mol BW (2008). Predictive value of pregnancy history in subfertile couples: results from a nationwide cohort study in the Netherlands. Fertil Steril.

[42] Vaegter KK, Lakic TG, Olovsson M, Berglund L, Brodin T, Holte J (2017). Which factors are most predictive for live birth after in vitro fertilization and intracytoplasmic sperm injection (IVF/ICSI) treatments? Analysis of 100 prospectively recorded variables in 8,400 IVF/ICSI single-embryo transfers. Fertil Steril.

[43] Nicoloro-SantaBarbara J, Busso C, Moyer A, Lobel M (2018). Just relax and you'll get pregnant? Meta-analysis examining women's emotional distress and the outcome of assisted reproductive technology. Soc Sci Med.

[44] Buysse DJ, Reynolds CF, Monk TH, Berman SR, Kupfer DJ (1989). The Pittsburgh sleep quality index: a new instrument for psychiatric practice and research. Psychiatry Res.

[45] Carpenter JS, Andrykowski MA (1998). Psychometric evaluation of the Pittsburgh sleep quality index. J Psychosom Res.

[46] Lintsen AM, Verhaak CM, Eijkemans MJ, Smeenk JM, Braat DD (2009). Anxiety and depression have no influence on the cancellation and pregnancy rates of a first IVF or ICSI treatment. Hum Reprod.

[47] Klonoff-Cohen H, Chu E, Natarajan L, Sieber W (2001). A prospective study of stress among women undergoing in vitro fertilization or gamete intrafallopian transfer. Fertil Steril.

[48] de Klerk C, Hunfeld JA, Heijnen EM, Eijkemans MJ, Fauser BC, Passchier J, Macklon NS (2008). Low negative affect prior to treatment is associated with a decreased chance of live birth from a first IVF cycle. Hum Reprod.

[49] Thiering P, Beaurepaire J, Jones M, Saunders D, Tennant C (1993). Mood state as a predictor of treatment outcome after in vitro fertilization/embryo transfer technology (IVF/ET). J Psychosom Res.

[50] Smeenk JM, Verhaak CM, Eugster A, van Minnen A, Zielhuis GA, Braat DD (2001). The effect of anxiety and depression on the outcome of in-vitro fertilization. Hum Reprod.

[51] Boivin J, Schmidt L (2005). Infertility-related stress in men and women predicts treatment outcome 1 year later. Fertil Steril.

[52] Verhaak CM, Smeenk JM, Nahuis MJ, Kremer JA, Braat DD (2007). Long-term psychological adjustment to IVF/ICSI treatment in women. Hum Reprod.

[53] Boivin J, Lancastle D (2010). Medical waiting periods: imminence, emotions and coping. Womens Health (Lond).

[54] Chen ZJ, Shi Y, Sun Y, Zhang B, Liang X, Cao Y, Yang J, Liu J, Wei D, Weng N (2016). Fresh versus frozen embryos for infertility in the polycystic ovary syndrome. N Engl J Med.

[55] Lintsen AM, Eijkemans MJ, Hunault CC, Bouwmans CA, Hakkaart L, Habbema JD, Braat DD (2007). Predicting ongoing pregnancy chances after IVF and ICSI: a national prospective study. Hum Reprod.

[56] Palmer CA, Alfano CA (2017). Sleep and emotion regulation: An organizing, integrative review. Sleep Med Rev.

[57] Huang LH, Kuo CP, Lu YC, Lee MS, Lee SH (2019). Association of emotional distress and quality of sleep among women receiving in-vitro fertilization treatment. Taiwan J Obstet Gynecol.

[58] Skouteris H, Wertheim EH, Germano C, Paxton SJ, Milgrom J (2009). Assessing sleep during pregnancy: a study across two time points examining the Pittsburgh sleep quality index and associations with depressive symptoms. Womens Health Issues.

[59] Baribeau DA, Dupuis A, Paton TA, Hammill C, Scherer SW, Schachar RJ, Arnold PD, Szatmari P, Nicolson R, Georgiades S (2019). Structural neuroimaging correlates of social deficits are similar in autism spectrum disorder and attention-deficit/hyperactivity disorder: analysis from the POND network. Transl Psychiatry.

