# Supplementary material for: Stress, anxiety, and depression in infertile couples are not associated with a first IVF or ICSI treatment outcome
Source: BMC Pregnancy Childbirth. 2021 Oct 27;21:725. doi: 10.1186/s12884-021-04202-9 (PMC8549180; doi:10.1186/s12884-021-04202-9)
Supplement: Supplementary file 1 — Additional file 1: Supplementary Table 1. Characteristics of the ART procedures among 450 couples undergoing their first recorded fresh IVF or ICSI cycle. Supplementary Table 2. Conditional logistic Regression Analysis of psychological distress level on pregnancy rate of first IVF or ICSI cycle among couples without endometriosis, or chronic endometritis or autoimmune disorders. Supplementary Table 3. Conditional logistic Regression Analysis of psychological distress symptoms on pregnancy rate of first IVF or ICSI cycle among women without endometriosis, or chronic endometritis or autoimmune disorders. Supplementary Figure 1. Flowchart for inclusion and exclusion of the study population. [file 12884_2021_4202_MOESM1_ESM.docx]

**Stress, anxiety, and depression in infertile couples are not associated with a first IVF** **or ICSI treatment outcome**

Meijuan Peng^1,2†^, Mingyang Wen^1,2 †^, Tao Jiang^2,3†^, Yangqian Jiang^1,2^, Hong Lv^1,2^, Ting Chen^4^, Xiufeng Ling^2,5^, Hong Li^2,6^, Qingxia Meng^2,6^, Boxian Huang^2,6^, Shiyao Tao^1,2^, Lei Huang^1,2^, Cong Liu ^1,2^, Xin Xu^1,2^, Qun Lu^1,2^, Xiaoyu Liu^1,2^, Bo Xu^1,2^, Xiumei Han^1,2^, Kun Zhou^1,2^, Jiaping Chen^1,2^, Yuan Lin^1,2,7^, Hongxia Ma^1,2^, Yankai Xia^1,8^, Hongbing Shen^1,2^, Zhibin Hu^1,2^, Feng Chen ^2,3^, Jiangbo Du^1,2*^, Guangfu Jin^1,2*^

1. Department of Epidemiology, Center for Global Health, School of Public Health, Nanjing Medical University, Nanjing 211166, Jiangsu, China

2. State Key Laboratory of Reproductive Medicine, Nanjing Medical University, Nanjing 211166, Jiangsu, China

3. Department of Biostatistics, School of Public Health, Nanjing Medical University, Nanjing 211166, Jiangsu, China

4. Department of Science and Technology, Women's Hospital of Nanjing Medical University, Nanjing Maternity and Child Health Care Hospital, Nanjing 210004, Jiangsu, China

5. Department of Reproduction, Women's Hospital of Nanjing Medical University, Nanjing Maternity and Child Health Care Hospital, Nanjing 210004, Jiangsu, China

6. Reproductive Genetic Center, The Affiliated Suzhou Hospital of Nanjing Medical University, Suzhou Municipal Hospital, Gusu School, Nanjing Medical University, Suzhou 215002, Jiangsu, China

7. Department of Maternal, Child and Adolescent Health, School of Public Health, Nanjing Medical University, Nanjing 211166, Jiangsu, China

8. Key Laboratory of Modern Toxicology of Ministry of Education, School of Public Health, Nanjing Medical University, Nanjing 211166, Jiangsu, China

† Peng M and Wen M, and Jiang T contribute equally to this work.

🖂Correspondence to:

Jiangbo Du, E-mail: dujiangbo@njmu.edu.cn, Department of Epidemiology, Center for Global Health, School of Public Health, Nanjing Medical University, Nanjing 211166, Jiangsu, China; or Guangfu Jin, E-mail: guangfujin@njmu.edu.cn, Department of Epidemiology, Center for Global Health, School of Public Health, Nanjing Medical University, Nanjing 211166, Jiangsu, China

**Supplementary information**

**Supplementary Table 1.** Characteristics of the ART procedures among 450 couples undergoing their first recorded fresh IVF or ICSI cycle.

**Supplementary Table 2.** Conditional logistic Regression Analysis of psychological distress level on pregnancy rate of first IVF or ICSI cycle among couples without endometriosis, or chronic endometritis or autoimmune disorders

**Supplementary Table 3.** Conditional logistic Regression Analysis of psychological distress symptoms on pregnancy rate of first IVF or ICSI cycle among women without endometriosis, or chronic endometritis or autoimmune disorders.

**Supplementary Figure 1.** Flowchart for inclusion and exclusion of the study population.

**Supplementary Table 1.** Characteristics of the ART procedures among 450 couples undergoing their first recorded fresh IVF or ICSI cycle.

| **Covariate** | **Pregnancy  N=300** | **Non-pregnancy  N=150** | ***P*** |
| --- | --- | --- | --- |
| **Fertilization method (%)**^d^ |  |  |  |
| IVF | 218 (72.7) | 103 (68.7) | 0.171^b^ |
| ICSI | 78 (26.0) | 46 (30.7) |  |
| IVF+ICSI | 4 (1.3) | 0 (0.0) |  |
| **Type of infertility (%)** |  |  |  |
| Secondary | 125 (41.7) | 68 (45.3) | 0.522^b^ |
| Primary | 175 (58.3) | 82 (54.7) |  |
| **Treatment cycle protocols (%)** |  |  |  |
| Agonist protocol | 189 (63.0) | 96 (64.0) | 0.709^c^ |
| Antagonist protocol | 108 (36.0) | 54 (36.0) |  |
| Other | 3 (1.0) | 0 (0.0) |  |
| **No. of embryos transferred (%)** |  |  |  |
| 1 | 70 (23.3) | 48 (32.0) | 0.071^c^ |
| 2 | 224 (74.7) | 97 (64.7) |  |
| 3 | 6 (2.0) | 5 (3.3) |  |
| **Stage of embryo transferred (%)** |  |  |  |
| Blastocyst | 41 (13.7) | 24 (16.0) | 0.602^b^ |
| Cleavage | 259 (86.3) | 126 (84.0) |  |
| **Endometrial thickness on hCG trigger day (mm)** ^d^ |  |  |  |
|  | 10.4 (8.9, 11.9) | 10.1 (8.7, 11.6) | 0.380^a^ |
| **No. of good-quality embryos transferred (%)** |  |  |  |
| 0 | 26 (8.7) | 18 (12.0) | 0.249^b^ |
| 1 | 73 (24.3) | 43 (28.7) |  |
| ≥2 | 201 (67.0) | 89 (59.3) |  |
| **Embryo sum score** |  |  |  |
|  | 6.0 (4.0, 6.0) | 6.0 (3.0, 6.0) | 0.094^a^ |
| **Basal hormones**^d^ |  |  |  |
| Basal Follicle-stimulating hormone — IU/liter | 7.0 (6.0, 8.3) | 7.3 (6.3, 8.5) | 0.172^a^ |
| Basal Estradiol — pg/ml | 44.0 (32.5, 57.0) | 44.0 (34.0, 56.6) | 0.572^a^ |
| Basal progesterone — ng/mL | 0.7 (0.5, 1.0) | 0.8 (0.5, 1.4) | 0.086^a^ |
| Basal Prolactin — ng/ml | 14.4 (11.1, 19.6) | 14.9 (11.1, 18.7) | 0.852^a^ |
| Basal Luteinizing hormone — IU/liter | 4.2 (3.2, 5.6) | 4.5 (3.5, 5.8) | 0.263^a^ |
| Basal Total testosterone — ng/ml | 0.5 (0.4, 0.6) | 0.5 (0.4, 0.6) | 0.694^a^ |
| Basal Anti-mullerian hormone — ng/mL | 3.3 (2.2, 4.6) | 3.5 (2.0, 4.7) | 0.973^a^ |
| Values are number of patients (%) for categorical variables, median and range for continuous variables unless indicated otherwise. | | | |
| ^a^ *P* values were derived with Mann–Whitney U test for nonnormally distributed continuous variables.  ^b^ *P* values were derived with Pearson chi-square test for categorical variables.  ^c^ *P* values were derived with Fisher exact test for categorical variables with less than 10 observations per category.  ^d^ Variable contains missing data. | | | |

**Supplementary Table 2.** Conditional logistic Regression Analysis of psychological distress level on pregnancy rate of first IVF or ICSI cycle among couples without endometriosis, or chronic endometritis or autoimmune disorders

| **Mood parameters** | **Pregnancy** | **Non-pregnancy** | ***P^a^*** | **Crude OR^b^** | **Adjusted OR^c^ (95% CI)** | **Adjusted OR^d^ (95%CI)** |
| --- | --- | --- | --- | --- | --- | --- |
|  |  |  |  | **(95% CI)** |  |  |
| **SAS score (anxiety)** |  |  |  |  |  |  |
| Women | 31.00 (26.00, 36.00) | 32.50 (26.75, 37.00) | 0.294 | 0.99 (0.95-1.04) | 0.99 (0.94-1.04) | 0.98 (0.92-1.04) |
| Partner^e^ | 28.00 (25.00, 33.00) | 28.00 (26.00, 33.00) | 0.544 | 0.99 (0.94-1.05) | 0.97 (0.91-1.04) | 0.98 (0.91-1.06) |
| Couple^e^ | 61.00 (53.00, 68.00) | 62.00 (55.00, 69.00) | 0.686 | 0.99 (0.96-1.02) | 0.98 (0.94-1.02) | 0.98 (0.94-1.02) |
| **CESD score (depression)** |  |  |  |  |  |  |
| Women | 6.00 (2.00, 12.00) | 6.50 (2.75, 11.00) | 0.956 | 0.98 (0.94-1.04) | 0.98 (0.93-1.03) | 0.97 (0.92-1.03) |
| Partner^e^ | 4.00 (0.00, 9.00) | 5.00 (1.00, 10.00) | 0.239 | 1.00 (0.95-1.05) | 0.99 (0.93-1.04) | 0.98 (0.92-1.05) |
| Couple^e^ | 12.00 (3.00, 20.00) | 13.00 (6.00, 21.75) | 0.631 | 0.99 (0.97-1.03) | 0.99 (0.95-1.02) | 0.98 (0.94-1.02) |
| **PSS score (perceived stress)** |  |  |  |  |  |  |
| Women | 11.00 (4.00, 15.50) | 11.00 (6.00, 14.25) | 0.949 | 1.00 (0.95-1.04) | 1.00 (0.95-1.05) | 1.00 (0.94-1.05) |
| Partner^e^ | 10.00 (4.00, 14.00) | 9.00 (3.00, 15.00) | 0.928 | 0.97 (0.92-1.02) | 0.95 (0.90-1.00) | 0.97 (0.91-1.03) |
| Couple^e^ | 23.00 (8.00, 29.00) | 20.00 (10.00, 27.00) | 0.6 | 0.98 (0.96-1.01) | 0.98 (0.95-1.01) | 0.98 (0.95-1.02) |
| **SAS+CESD+PSS (composite psychological distress)** |  |  |  |  |  |  |
| Women | 48.00 (34.00, 63.00) | 51.00 (37.75, 62.00) | 0.671 | 1.00 (0.98-1.01) | 1.00 (0.98-1.01) | 0.99 (0.97-1.01) |
| Partner^e^ | 43.00 (32.00, 56.00) | 42.50 (33.25, 56.75) | 0.666 | 1.00 (0.98-1.02) | 0.99 (0.96-1.01) | 0.99 (0.96-1.01) |
| Couple^e^ | 97.00 (64.50, 115.75) | 97.00 (73.50, 116.25) | 0.838 | 1.00 (0.99-1.01) | 0.99 (0.98-1.00) | 0.99 (0.98-1.01) |
| Values are median and range for continuous variables unless indicated otherwise. | | | | | | |
| ^a^ *P* values were derived with Mann-Whitney U test for nonnormally distributed continuous variables.  ^b^ Univariable conditional logistic regression analyses of psychological distress level on pregnancy rate of first IVF or ICSI cycle.  ^c^ Model1: Multivariable conditional logistic regression analyses were adjusted for female pre-treatment BMI, educational attainment, occupation, household income, infertility factor, duration of infertility.  ^d^ Model2: Multivariable conditional logistic regression analyses were adjusted for female pre-treatment BMI, educational attainment, occupation, household income, infertility factor, duration of infertility, prior history of pregnancy loss, alcohol use, sleep quality, exercise, female and male smoking before the start of treatment.  ^e^ Variable contains missing data. | | | | | | |

**Supplementary Table 3.** Conditional logistic Regression Analysis of psychological distress symptoms on pregnancy rate of first IVF or ICSI cycle among women without endometriosis, or chronic endometritis or autoimmune disorders.

| **Mood parameters** | **N** | **No. of  non-pregnancy (%)** | **Crude OR^a^ (95%CI)** | **Adjusted OR^b^ (95%CI)** | **Adjusted OR^c^ (95%CI)** |
| --- | --- | --- | --- | --- | --- |
| **Depression** |  |  |  |  |  |
| No | 211 | 72 (34.1) | 1 | 1 | 1 |
| Yes | 36 | 12 (33.3) | 0.88 (0.33-2.38) | 0.79 (0.28-2.27) | 0.50 (0.15-1.64) |
| **Anxiety** |  |  |  |  |  |
| No | 241 | 84 (34.9) | 1 | 1 | 1 |
| Yes | 6 | 0 (0.0) | - | - | - |
| ^a^ Univariable conditional logistic regression analyses of psychological distress level on pregnancy rate of first IVF or ICSI cycle.  ^b^ Model1: Multivariable conditional logistic regression analyses were adjusted for female pre-treatment BMI, educational attainment, occupation, household income, infertility factor, duration of infertility.  ^c^ Model2: Multivariable conditional logistic regression analyses were adjusted for female pre-treatment BMI, educational attainment, occupation, household income, infertility factor, duration of infertility, prior history of pregnancy loss, alcohol use, sleep quality, exercise, female and male smoking before the start of treatment. | | | | | |


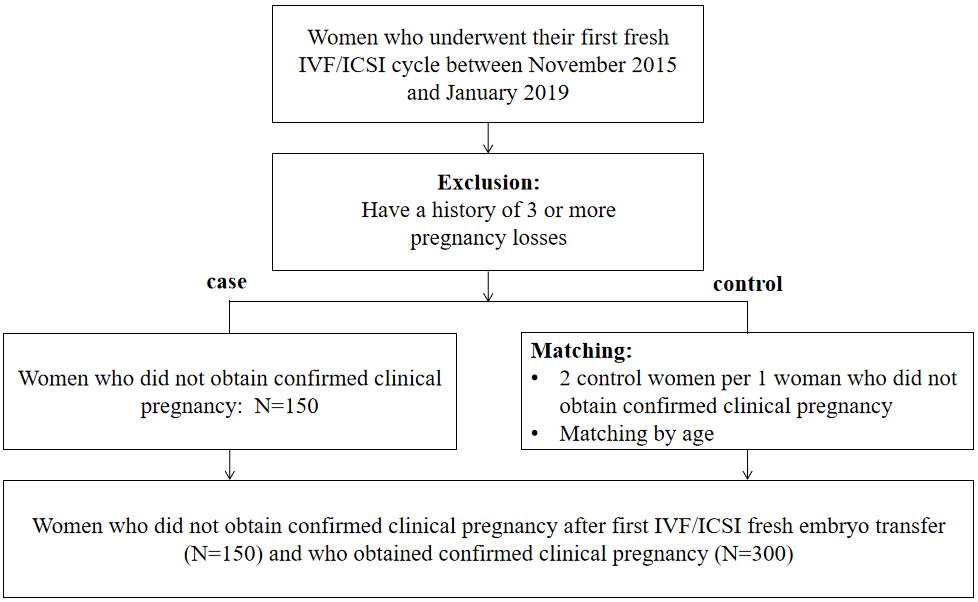


**Supplementary Figure 1.** Flowchart for inclusion and exclusion of the study population.
